# Improving performance in golf: current research and implications from a clinical perspective

**DOI:** 10.1590/bjpt-rbf.2014.0122

**Published:** 2015-10-06

**Authors:** Kerrie Evans, Neil Tuttle

**Affiliations:** 1 School of Allied Health Sciences, Menzies Health Institute Queensland, Griffith University, Gold Coast campus, Queensland, Australia

**Keywords:** golf swing, kinematics, exercise programs, movement variability, biomechanics

## Abstract

Golf, a global sport enjoyed by people of all ages and abilities, involves relatively long periods of low intensity exercise interspersed with short bursts of high intensity activity. To meet the physical demands of full swing shots and the mental and physical demands of putting and walking the course, it is frequently recommended that golfers undertake golf-specific exercise programs. Biomechanics, motor learning, and motor control research has increased the understanding of the physical requirements of the game, and using this knowledge, exercise programs aimed at improving golf performance have been developed. However, while it is generally accepted that an exercise program can improve a golfer's physical measurements and some golf performance variables, translating the findings from research into clinical practice to optimise an individual golfer's performance remains challenging. This paper discusses how biomechanical and motor control research has informed current practice and discusses how emerging sophisticated tools and research designs may better assist golfers improve their performance.

## Introduction

The inclusion of golf in the 2016 Summer Olympic Games for the first time since 1904 is an indicator of the increasing globalisation of the sport. It is estimated that worldwide between 55 and 80 million people from at least 136 countries play golf^1 3^, with the more avid golfers playing more than once a week, every week of the year. The vast majority of people who play golf are amateur golfers, with only a very small proportion being considered elite amateurs and fewer still are professional golfers. Irrespective of whether a golfer is an amateur or a professional, the goal is the same - to complete a round of golf in as few strokes (shots) as possible and, from a longevity perspective, continue to enjoy the game as pain and injury free as possible.

### The game of golf

Golf is a sport that involves a relatively long duration of low intensity activity interspersed with short bursts of high intensity activity. Golf courses vary in length and terrain, so a round of 18 holes can take between 3.5 and 6 hours to play and, if the players are walking, results in a low moderate intensity form of aerobic exercise^4^^,^ 5. However, as much as 60% of the time taken to play a round of golf is spent preparing and performing swings, and of this time, 25% is spent putting on the green^6^. In contrast to the relatively low intensity demand of the rest of the game, a full swing action requires a rapid expenditure of energy. For example, professional golfers perform a swing with a driver in 1.09 seconds^7^, with the club head reaching speeds of more than 160 km/hour^8^. Overall muscle activity when using a 5 iron reaches 90% of maximal voluntary contraction (MVC) for amateurs and 80% for professionals^9^, and golfers perform an average of 30 40 swings every round with these high levels of intensity^10^. In contrast to full swings, the putting stroke requires minimal body movement but involves the greatest degree of sustained trunk inclination and sagittal flexion compared with shots with other clubs^6^. It has been suggested that, particularly when practised for prolonged periods, putting may challenge a golfer's postural endurance^11^^,^ 12. Researchers and clinicians wanting to optimise performance and prevent golf injury have hypothesised that specific golf exercise programs are necessary to meet the physical demands of both full swing shots and the potential fatigue associated with putting or walking^13^^,^ 14.

### Biomechanical investigations of the golf swing

The landmark work of Cochran and Stobbs^15^in 1968 employed high-speed filming techniques to examine the components of the golf swing, ball aerodynamics, and equipment dynamics. Since then, there has been a vast range of biomechanical studies that have examined the highly complex, multi joint movements involved in the golf swing. Researchers have used 2D and 3D methods, including high speed video^16^, optoelectronic^12^^,^ 17 ^-^ 19 and electromagnetic motion tracking systems^20^^,^ 21, computer modelling^22^, force plates^23^^-^ 25, wireless inertial sensors^26^, and electromyography^27 31^ to gain insight into and quantify the fundamental elements of the swing. The majority of studies have been conducted in laboratory settings and most have employed indirect measures of golf performance such as club head velocity (CHV) and ball launch characteristics^18^^,^ 23 ^,^ 32 ^,^ 33. Laboratory based studies have clear advantages, including ease of standardisation, greater environmental control, and the degree of accuracy possible with some indoor motion analysis systems. On the other hand, swinging a golf club indoors surrounded by expensive equipment may not reflect what happens on the golf course, and there is concern that the indirect measures of performance used in laboratory conditions may provide incomplete information about actual golf performance. Some studies have been conducted outdoors and on golf courses^6^^,^ 34; however, more research is needed to examine how golfers perform their swing on the course, over a round of golf, and under competition conditions and how these findings relate to what occurs in laboratory settings. Not only will these types of studies provide ecologically valid biomechanical information, but they will also provide more specific information about the physical demands of the sport and how environmental or other factors, such as pressure or fatigue, affect golf performance.

Due to the importance of the full swing, particularly in driving performance^32^, and perhaps because of the fact that this stroke could be considered as having the most repeatable intention to hit the ball as far and straight as possible most kinematic studies have concentrated on full swing kinematics. In spite of the golf swing being dynamic by nature, many of these studies have measured parameters (e.g. segmental orientation) at discrete time points during the swing, such as address, top of backswing, ball contact. Collectively, findings have provided valuable insights into, for example, the magnitude of thorax and pelvis movement when high CHV are produced^7^^,^ 35 ^,^ 36, differences in segmental angular velocities between skilled and less skilled golfers^37^^,^ 38, and the importance of the magnitude, sequencing, and timing of segmental motion^35^^,^ 39 ^,^ 40. The results have helped inform research investigating physical characteristics required for skilled golf performance.

With the increasing awareness of the importance of movement variability in skilled performance^41 43^, there has been growing interest in investigating the complex segment and intersegmental coordination that occurs during the full swing^44 47^. Movement variability can be described as the normal variations that occur in motor performance across multiple repetitions of a task^48^. Historically, movement variability observed in skilled sporting tasks was considered "noise" or error and therefore undesirable. It is now recognised that variability has a functional role and does not necessarily result in outcome variability^41^^,^ 45 ^,^ 49. That is, there is greater understanding of the large number of constraints that interact to shape movement behaviours during sporting endeavours, including body properties, environmental conditions, and tasks, and that highly skilled performers demonstrate the necessary flexibility and adaptability to operate proficiently in a variety of learning and performance contexts^42^^,^ 50.

Movement variability in the downswing of skilled male and female golfers was investigated by Horan et al.^51^. Despite variability in the kinematics of the thorax and pelvis as well as variability in thorax pelvis coupling at the midpoint of the downswing and at ball contact, both males and females achieved highly consistent club and hand trajectories at ball contact. Interestingly, females were found to have greater variability in thorax pelvis coupling than males. While physiological measures were not directly measured, the differences may have been due to differences in factors such as strength or flexibility or that male and female golfers adopted different motor control strategies to achieve consistent performance. Gender related differences in golf swing kinematics have been observed by other authors^38^^,^ 39 ^,^ 52 supporting the notion that a number of characteristics will influence a golfer's pattern of movement and coordinative strategies.

**Figure 1.**
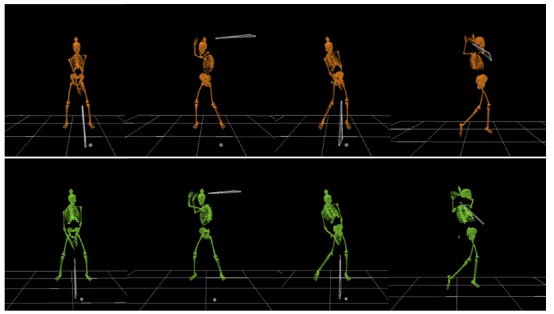
Full swing by two golfers demonstrating between individual variations. From left: address position, top of backswing, impact, and follow through.

**Figure 2:**
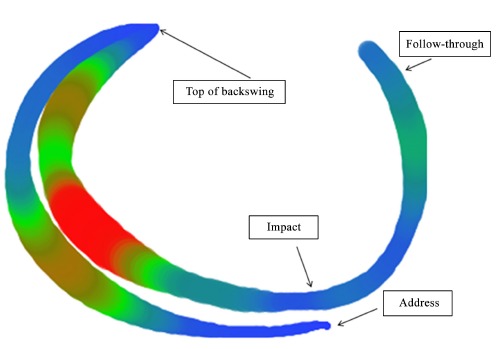
The 3D trajectory of the club head of one golfer performing multiple swings demonstrating within individual variation. The width and colour of the pathway indicate the magnitude and direction of variability. The width at the point of impact is narrower indicating considerably less variability than the backswing and downswing that precede it.

The concept that movement variability in individual segmental trajectories during a specific task may not be detrimental to outcome performance as long as the critical 'end point parameters' (in the case of the golf swing, club head parameters at ball contact) remain consistent^49^^,^ 53 was supported more recently by Tucker et al.^54^. These authors found that a group of highly skilled golfers maintained consistency of ball speed despite variability in movement of individual body segments during the swing. Variability of movement of the individual body segments are integrated to produce a reduced variability in the club head trajectory, which in turn results in an even smaller variability in the club head on contact with the ball. Additionally, Tucker et al.^54^ found that movement variability was highly individual-specific with different golfers adopting different performance strategies to preserve shot outcome. Taken collectively, emerging evidence supports the notions of 1) inter player variability, i.e. that individual golfers have individualised swing patterns that are different from the patterns of other golfers (Figure 1), and 2) intra player variability, i.e. that within their own swing pattern, each individual has variation in the contributions from the many different components (Figure 2).

### Clinical implications

Golf has been described as one of the most complex, technically demanding and high precision sports that exist^55^. Clinicians that work with golfers should consider that inter golfer and intra golfer variability in swing performance will be affected by task, environment, and organism constraints, all of which interact to determine the patterns of motion that are observed when a golfer swings a club^45^. Despite an increased understanding of the swing from both biomechanics and neuroscience research, the best way to optimise both swing and outcome performance for an individual golfer remains elusive. From a physical therapist's perspective, optimising performance in golf requires knowledge of not only the technical and physical requirements of the sport, but also how these domains are interrelated with the fields of psychology, motor learning, and motor control. While recognising the importance of a multimodal approach to optimising golf performance, the following sections focus on the physical requirements of golf and evidence pertaining to whether exercise programs can help golfers improve their performance.

### Physical requirements of the golf swing

Highly skilled golfers tend to have different physical characteristics than less proficient golfers^56^ and factors such age, gender, and history of injury also influence a golfer's performance on physical tests as well as swing parameters^39^^,^ 57 ^,^ 58. Nevertheless, a combination of mobility, stability, strength, and cardiovascular fitness is frequently recommended for optimal 'golf fitness'^14^^,^ 59. Kinematic studies have highlighted the importance of adequate flexibility, particularly in the trunk, hips, and shoulders, to achieve the body positions required to optimise CHV^52^^,^ 56 ^,^ 60. For example, reported averages for torso rotation during the backswing for a driver range from 78° to 109° with the pelvis rotating to a lesser extent of between 37° and 64°^7^^,^ 35 ^,^ 52. EMG studies have sought to identify the muscle groups important for golf performance^28,29,61 64^ and several reviews have been published on this topic^65^^,^ 66. From the collated data, it is apparent that the trunk extensors, hip extensors, and the abdominal muscles all play an important role in producing a powerful efficient golf swing. The efficient transfer of energy from the lower body to the muscle groups of the chest and arms and eventually the hands and club the "bottom up phenomenon"^60^ is important for producing high CHV, but similarly to swing kinematics, a number of kinetic variables measured during the swing are also highly individual-specific^22^.

Golfers spend many hours practising. Professional golfers can perform up to 300 swings in a single practice session and hit over 2000 shots per week^67^^,^ 68. To ensure a golfer can meet both the physical and mental demands of playing tournament golf and avoid the detrimental effects that fatigue has been shown to have on performance^11^^,^ 69, exercises aimed at improving a golfer's cardiovascular fitness have also been advocated^14^.

In summary, playing golf has very specific physical requirements that have led many researchers, coaches, and clinicians to suggest that physical preparation programs should be undertaken by golfers of all ages and abilities in order to improve performance and prevent golf related injury. This paper will not focus on the latter but on findings from studies that have investigated whether exercise programs can improve golf performance.

### Exercise programs to improve golf performance

Golf-specific exercises have been advocated for many years, with early attempts being largely idiosyncratic and based on personal experience and opinion. For example, three-time Open Championship winner Sir Henry Cotton in 1948 said:

Let me add, that, as far as I know, no data on this subject of specific golf muscle-building has ever been given, and I have had to grope my way along according to my own ideas and following my own observations, endeavouring to build up my golfing muscles to the best of my ability^70^.

Cotton's statement reflects the predominant understanding of human performance in the 1940's: increased muscular strength should result in improved performance. A golf specific exercise program would therefore be designed to target the specific muscles used in the sport. In their review of strength and conditioning programs for improving fitness in golfers, Smith et al.^71^ defined golf-specific exercises as those that activate muscles groups that are used in golf in comparable patterns of motor coordination, in similar planes and ranges of movements, with similar speeds, and similar loads on postural muscles. In addition to load, this definition adds coordination, pattern specificity, and speed to the idea of what makes exercises golf-specific. Interestingly, Smith et al.^71^ concluded that the majority of studies included in their review involved reasonably generic exercise programs that did not fulfil the criteria for being golf-specific. The exercises employed ranged from free weights and medicine ball plyometric training in young male golfers (age: 29±7.4 yrs, handicap: 5.5±3.7)^72^ to strength and flexibility exercises in older recreational golfers (age: 65.1±6.2 yrs of all skill levels)^73^ to a proprioceptive neuromuscular facilitation stretching program in golfers aged between 47 and 82 years with handicaps ranging from 8 to 34^74^. Despite the fact that several of the studies reviewed by Smith et al.^71^ had low methodological scores, it is nevertheless interesting to see that, seemingly irrespective of the type of exercise approach, the duration of the program, the age or skill of the golfer, the majority of studies reported improvements in at least some of the fitness (e.g. muscular strength, flexibility) and golf performance variables (e.g. club head speed, driving distance) that were measured.

Since Smith et al.'s^71^ 2011 review, as well as that of Torres Ronda et al.^75^, further studies have investigated the effects of different exercise approaches on parameters, such as club head speed, ball spin, and swing kinematic variables, thought to relate to golf performance. These studies have again been diverse in terms of the exercises prescribed (e.g. 'isolated core training'^76^, plyometric training^77^, combination of maximal strength, plyometric and golf-specific exercises^78^, different warm up programs^79^); duration of the program (range 6 weeks^80^ to 18 weeks^78^); age and skill level of the golfers (e.g. ~24 years with handicap <5^80^ vs ~47 years with a mean handicap of 11.2±6.1^78^); effect sizes; and methodological quality. Similar to previous work, direct measures of golf performance (e.g. strokes per round, performance during tournaments) are lacking. Overall, the results support the notion that it is more important that a golfer do *some form of exercise* rather than no exercise, irrespective of what particular type of exercise is undertaken.

### Lessons from other areas of clinical research

Interestingly, the conclusion that exercise (generally) has a beneficial effect for golfers, regardless of the type of exercise, is similar to findings in other areas of sports research^81^^,^ 82 but most notably the low back pain (LBP) field. Historically, most reviews of exercise therapy for patients with LBP conclude that when different types of exercise are compared directly, exercise in general is effective^83 85^. That is, there does not appear to be one form of exercise that is superior to another for patients with LBP. What the studies do not tell us, however, by reporting group means, is whether one program is better for a given individual and if so, which one. More recently, studies comparing interventions based on subgrouping of patients and development of clinical prediction rules have been conducted with the aim of more specifically tailoring interventions based on a set of patient characteristics. However, it has proven extremely challenging to develop theoretical and practical frameworks that consider enough of a patient's biological as well as psychosocial characteristics to determine effective treatment strategies^86^. Nevertheless, there is preliminary evidence supporting the notion that patients who receive a more individualised treatment approach achieve better outcomes^87^.

To date, when studies of the effects of exercise programs on golf performance have subgrouped participants, the grouping criteria have been according to handicap, age, or gender. Grouping a golfer based on handicap intuitively makes the most sense - skilled golfers have more consistent swing kinematics than unskilled golfers and therefore any changes post intervention are more likely to be as a result of the intervention than due to measurement error. However, one only has to look at the player anthropometrics of the Ladies Professional Golf Association's (LPGA) Top 10 female golfers to recognise that even the best players in the world are reasonably heterogeneous.

### Where to from here?

There is still much to understand about how to assist golfers improve their game and avoid injury. It will be important to ensure the validity of the measurements that are being made, consider more sophisticated measures or methods of analysis, and ensure that the outcomes being considered are true indicators of the desired outcomes. Perhaps most importantly, however, is to use measures that reflect the dynamic nature of golf and are capable of taking into consideration individual variation in strategies and responses.

New tools such as a variety of wearable sensors, marker-less motion tracking, and wide field-of-view electromagnetic tracking systems are becoming available that can assist to improve our understanding of the biomechanics and by enabling studies to be carried out on the golf course instead of the laboratory. Alternatively, if laboratory studies continue to be used, it will be important to cross validate the methodologies to ensure what occurs in the lab actually reflects what occurs on the course. Similarly, it will be important to determine how the surrogate measures of performance typically used in the lab relate to performance on the course.

The systems that are currently used in most biomechanics laboratories are able to determine location of points on the body and ground reaction forces at rates of hundreds or even thousands of samples per second and create a 3D reconstruction of the entire movement pattern through time. In spite of the dazzling complexity and accuracy of the data, much of the analyses use simplified variables such as maximum or minimum values of locations, angles, speeds, or accelerations or the values of these parameters at predetermined time points during the swing. One of a relatively small number of studies that evaluated data across the time course of the swing was that of Tucker et al.^54^. The authors recorded the locations of 14 points on the golfer's body and club at 400 Hz for 10 swings by each of 16 golfers. For each normalised time point for each marker, a virtual three dimensional ellipsoid was constructed that would contain the mean location +/ one standard deviation of the position of that marker through the swing. Not only does this type of methodology enable the swings of different individuals to be compared in ways that were not previously possible, but it also enables investigators to evaluate the relative impact of different body locations and/or time points on performance.

As more is understood about individual variation, it may be possible to develop and assess the efficacy of individualised programs for individual golfers. Instead of the more common study design, which compares two (or more) groups and have every member of the group receiving the same intervention, individualised programs could be assessed using a parallel group design. For example, the intervention in one group can be individualised according to an algorithm while the other intervention uses a set protocol^87^. Perhaps more appropriate, however, to evaluate individual treatment responses would be the use of so called "n of one trials"^88^. The power of this design comes from each intervention option being trialled more than once in a multiple crossover design (e.g. as a minimum - an ABAB or ABBA sequence). One type of intervention being consistently superior in more than one comparison provides much stronger evidence for it being actually superior. An advantage of n of one trials is that they are also available to the therapist in clinical practice. Consider for example if two exercise programs have demonstrated benefits, but in a head to head comparison neither is superior. 

One interpretation of the evidence would be to select one and only change the program if the outcomes were 'very poor'^89^. However, by applying an n of one design in clinical practice, the therapist no longer has to rely on average results but can determine which of the options is better for each individual golfer at a given time.

## Conclusions

Despite the growing body of research investigating the golf swing, much remains unknown and translating the findings from the biomechanical, physiological, motor learning, and motor control research into clinical practice, where the aim is to assist golfers improve their performance and prevent injury, remains challenging. It is generally well accepted that, in order to improve performance, a multimodal approach is required and both researchers and clinicians need to consider the aforementioned inter related dimensions in order to help optimise golf performance. There are general principles of exercise that are likely to be of benefit to all golfers, and the study designs employed to date have provided a wealth of information and should inform current and future practice. However, more sophisticated tools and designs are available that are capable of expanding our knowledge of golf and practice, thereby potentially increasing our ability to assist our clients improve their golf performance.
